# Quantitative Forecasting of Malaria Parasite Using Machine Learning Models: MLR, ANN, ANFIS and Random Forest

**DOI:** 10.3390/diagnostics14040385

**Published:** 2024-02-09

**Authors:** Dilber Uzun Ozsahin, Basil Barth Duwa, Ilker Ozsahin, Berna Uzun

**Affiliations:** 1 Department of Medical Diagnostic Imaging, College of Health Science, University of Sharjah, Sharjah 27272, United Arab Emirates; 2 Research Institute for Medical and Health Sciences, University of Sharjah, Sharjah 27272, United Arab Emirates; 3 Operational Research Centre in Healthcare, Near East University, TRNC Mersin 10, Nicosia 99138, Turkey; basil.barthduwa@neu.edu.tr (B.B.D.); ilker.ozsahin@neu.edu.tr (I.O.); 4 Brain Health Imaging Institute, Department of Radiology, Weill Cornell Medicine, New York, NY 10065, USA; 5 Department of Mathematics, Near East University, TRNC Mersin 10, Nicosia 99138, Turkey

**Keywords:** adaptive neuro-fuzzy inference system (ANFIS), artificial neural network (ANN), statistical prediction, malaria parasite, machine learning models, multiple linear regression (MLR)

## Abstract

Malaria continues to be a major barrier to socioeconomic development in Africa, where its death rate is over 90%. The predictive power of many machine learning models—such as multi-linear regression (MLR), artificial neural networks (ANN), adaptive neuro-fuzzy inference systems (ANFISs) and Random Forest classifier—is investigated in this study using data from 2207 patients. The dataset was reduced from the initial dataset of thirty-two criteria samples to fifteen. Assessment measures such as the root mean square error (RMSE), mean square error (MSE), coefficient of determination (*R*^2^), and adjusted correlation coefficient R were used. ANFIS, Random Forest, MLR, and ANN are among the models. After training, ANN outperforms ANFIS (97%), MLR (92%), and Random Forest (68%) with the greatest *R* (99%) and *R*^2^ (99%), respectively. The testing stage confirms the superiority of ANN. The paper also presents a statistical forecasting sheet with few errors and excellent accuracy for MLR models. When the models are assessed with Random Forest, the latter shows the least results, thus broadening the modeling techniques and offering significant insights into the prediction of malaria and healthcare decision making. The outcomes of using machine learning models for precise and efficient illness prediction add to an expanding body of knowledge, assisting healthcare systems in making better decisions and allocating resources more effectively.

## 1. Introduction

Malaria, also referred to as “fivre des marais” in French, is a tropical infectious illness transmitted by the parasite Plasmodium [1]. As reported by the World Health Organization (WHO), the annual mortality rate of malaria is roughly 435,000 globally, with sub-Saharan Africa bearing the brunt of the toll [2]. Malaria is particularly dangerous for children under the age of five, and it is responsible for a considerable amount of child mortality in Africa. Approximately 247 million infections caused by malaria were recorded in 2022 [3]. Mosquitoes (female anopheles) disseminate this disease through biting. The symptoms of malaria often develop 7–30 days following the infected mosquito bite and might include a high body temperature, a headache, muscular discomfort, and flu-like symptoms. Malaria can cause consequences such as anemia, renal failure, dyspnea, and cerebral malaria (a kind of severe malaria that affects the brain) in severe instances [4].

Malaria is among the most common infections in sub-Saharan regions. It thrives in subtropical areas, threatening public health. In other words, where health surveillance facilities are few, the impact is significant [5]. Therefore, an appropriate malaria forecasting framework is essential for lowering the detrimental effects of malaria prevalence in subtropical regions [6]. There have been increasing reports of the impacts of global warming, such as the increase and proliferation of insects that spread infection to people [7]. Numerous initiatives have been undertaken in recent years by governmental and nonprofit groups to completely eradicate malaria with the WHO being a prime example. Several studies have been conducted to either understand the disease from the perspective of the Plasmodium mosquito or to develop automated detection technologies [2]. In recent times, the epidemiology of malaria is revealed to be evolving. This is due to the overall number of traveling-related epidemics dropping concurrently, with the reduction in human activities experienced globally during the pandemic as a result of travel restrictions. In spite of this, multiple studies throughout the pandemic years warned of a potential rise for serious malaria among travelers coming from areas where malaria is prevalent [8].

Table 1 lists the nations most affected by malaria by the number of cases of malaria they have experienced for 2021 with Nigeria (94,000,000) as the highest and Tanzania (3,000,000) as the least. These numbers emphasize the serious impact malaria has on these nations and the urgent need for comprehensive malaria prevention and control methods. Additionally, the WHO predicted that 3.3 million malaria incidents would be reported annually worldwide [9]. A further 125 million pregnant women globally run the danger of contracting this disease annually. Up to 200,000 infant fatalities are attributed to maternal malaria each year in sub-Saharan Africa alone. Each year, there are around 10,000 cases of malaria in western Europe compared to 1300–1500 incidents in the United States [10].

Major decisions are influenced by forecasting. In order to plan and evaluate disease control, many strategies are employed to estimate future outcomes based on previous data. Forecasts provide information that consumers may use before making decisions or performing activities that may have an impact on the path of an epidemic. To predict epidemics, both linear and nonlinear models are utilized [11]. 

The research “A Review of Epidemic Forecasting Using Artificial Neural Networks” by Datilo et al. highlights the value of precision disease forecasts and suitable methods for making forecasts. They carried out a thorough analysis evaluating artificial neural networks (ANNs) to different forecasting techniques. The authors discovered that using hybrid models—ANNs combined with traditional methodologies or meta-heuristics, conversion strategies, and technology platforms—significantly improves the training and generalized capacities of ANNs in disease forecasting. The study finds that choosing the appropriate forecasting techniques is critical and suggests using ANN hybrids to make more precise and reliable forecasts about the scale of an outbreak [12]. Machine learning models are also adopted in other studies to predict, describe and diagnose medical conditions such as COVID-19 [13].

Multi-linear regression using GIS and remote sensing was used in mapping the spread of malaria in the Varanasi district of India. In the study conducted by [14], malaria cases reported in the research region served as the dependent variable, and multiple time-based groupings of average temperature data served as the independent factors. In the methodology, to create a malaria susceptibility map for both qualitative and quantitative variables, sampling of 50 × 50 was transferred from GIS to statistical software. However, in [14], the authors adopted both the GIS and statistical methods in their analysis. 

The advanced neuro-fuzzy inference system was also adopted in a study by [15] for the purpose of diagnosing malaria. Oladele et al. created the Coactive Neuro-Fuzzy Expert System. The tool boosts productivity and accuracy by combining fuzzy logic with neural networks. Oral interviews were used to record the expertise of medical professionals, which was then incorporated into the system’s knowledge base. Microsoft SQL Server 2012 and Microsoft Visual C# (C Sharp) were used to implement the software. Patients were given questionnaires to complete, which were then filled out by practitioners to record symptoms. The study showed how the neuro-fuzzy approach might be used in practice and concluded that DIAGMAL is a reliable malaria diagnosis tool [15].

Ozsahin et al. used peripheral blood smears to test deep learning frameworks for malaria parasite detection. Their target was to develop an accurate deep learning model, identify the optimal blood smear type, and compare the performance of their model to other transfer-learning methodologies. While using thick smears, their proposed convolutional neural network demonstrated accuracy, precision, and sensitivity of 96.97%, 97.00%, and 97.00%, respectively. This study underlines the need to select the appropriate smear type for improved accuracy and rapid detection in malaria-endemic locations [16].

Yadav et al. used clinical data to conduct a study on machine learning-based malaria prediction. The study intended to investigate and validate the efficacy of multiple machine-learning algorithms in predicting malaria based on clinical signs and symptoms. The study looked at two Senegal databases of malaria patients. The results demonstrated that Random Forest, Support Vector Machine with Gaussian Kernel, and artificial neural networks delivered promising and accurate results. On both datasets, these algorithms outperformed the Rapid Diagnostic Test, with accuracy, recall, and F1 scores of at least 92%, 85%, and 89%, respectively. Yadav et al. in their study demonstrate that machine learning algorithms can consistently detect the existence or the absence of malaria according to medical information [17].

Furthermore, choosing the best prediction model is critical for increased accuracy. Despite the fact that prior research has successfully used machine learning algorithms to reliably forecast other diseases, none have revealed the best suited model for malaria parasite prediction using the same models. The goal of this research is to develop a machine learning framework capable of effectively predicting malaria parasites based on laboratory symptoms. In addition, this study intends to determine which of the models was best suited for reliably predicting malaria parasites. Finally, we want to assess the performance of our proposed model using widely used evaluation measures such as *R*^2^, R, RMSE, and MSE.

## 2. Materials and Methods

### 2.1. Dataset

The original data were obtained from Kaggle.com as hematological data from 2207 patients in Ghana, as reported by Morang et al. [18]. The original dataset includes 32 criteria samples of 2207 cases. The criteria samples were reduced to 20. The dataset is characterized by 20 independent variables, which were subsequently reduced to 14 as the new variables to avoid overfitting. The variables include Fever Symptom, Temperature, Rapid Diagnostic Test (RDT), White Blood Cell Count (WBC), Red Blood Cell Count (RBC), Hemoglobin Level, Hematocrit, Mean Cell Volume, Mean Corp Hb, Mean Cell Hb Conc, Platelet Count, Platelet Distribution Width, Neutrophils Percent, Lymphocytes Percent, Mean Platelet Volume, Mixed Cells Percent, Neutrophils Count, Lymphocytes Count, Mixed Cells Count, RBC Distribution Width Percent, and Microscopy. The dependent variable or output is the microscopy, which is similar to the method implored by [19] in their study. Table 2 summarizes the details of the criteria and their values in binary 0 and 1. The study contains fifteen labeled features that are independent and one that is dependent.

### 2.2. Data Preprocessing

Data preprocessing is an imperative and typical initial step in any machine-learning modeling technique [20]. It allows raw data to be suitably prepared in network-acceptable forms. These procedures include data cleansing, which includes the identification and removal of unnecessary variables, as well as the normalization of the dataset [21]. In this study, the dataset was cleaned by identifying and removing unnecessary variables and columns, similar to [22,23]. The missing values were filled in by finding the column’s average value. The categorical dataset was changed to a numerical dataset, respectively [24]. The equation was applied as below:

(1) $$y=0.05+0.95\times (x-x_{min})/(x_{max}-x_{min})$$

where *x* is labeled as measured data, and $x_{min}$ and $x_{max}$ are the minimum and maximum points, respectively.

### 2.3. Machine Learning Prediction Models

#### 2.3.1. Advanced Neuro-Fuzzy Inference System (ANFIS)

The power of artificial neural networks and fuzzy reasoning are combined in a hybrid machine learning model known as adaptive neuro-fuzzy inference system (ANFIS). A learning method is used to adapt the fuzzy inference system, allowing it to detect complex relationships and generate accurate predictions [22]. ANFIS has the ability to recognize problems and locate solutions as they develop. Its predecessors were the feed-forward and multilayer adaptive networks. Input variables as well as input and output variables and the fuzzy rule collectively make up the ANFIS fuzzy rule, which is based on Takagi–Sugeno–Kan inferences and incorporates both independent and dependent variables [25]. The database of fuzzy includes both fuzzing and de-fuzzing. The information is transformed into fuzzified values via fuzzy set theory utilizing membership function parameters (MF). The MFs of the nodes played a crucial part in the modeling of the correlation involving the two parameters. Its constituent functions are triangular, trapezoid, and Gaussian. Equations (1) and (2) are developed based on the Takagi–Sugeno–Kan inferences.

(2) $$Rule\;No.1:\;if\;\mu \;(x)\;is\;A_{1}\;and\;\mu \;(y)\;is\;B_{1}\;then\;f_{1}\;=\;p_{1}x\;+\;q_{1}y\;+\;r_{1}$$

(3) $$Rule\;No.2:\;if\;\mu \;(x)\;is\;A_{2}\;and\;\mu \;(y)\;is\;B_{2}\;then\;f_{2}\;=\;p_{2}x\;+\;q_{2}y\;+\;r_{2}$$

The variables A_1_, B_1_, and B_2_ are the membership functions for x and y, whereas the inputs p_1_, q_1_, r_1_, and p_2_, q_2_, r_2_ provide the data for the output function. The ANFIS’s formulation and structure are compatible with a 5-tiered neural network design [26].

#### 2.3.2. Artificial Neural Network (ANN)

There are an overall total of fifteen layers that make up the replication type of ANN used in this study: fourteen input layers, fifteen hidden layers, as well as one output layer. Supervised training was used in the procedure, which used 70% of the total sample for training and thirty percent for testing. The target layer used a linear transfer function, and the hidden layer used a sigmoid activation function. Six epochs of training were conducted to show the neural network’s architecture and training parameters for successful malaria prediction, as shown in Table 3. 

**Figure 1:**
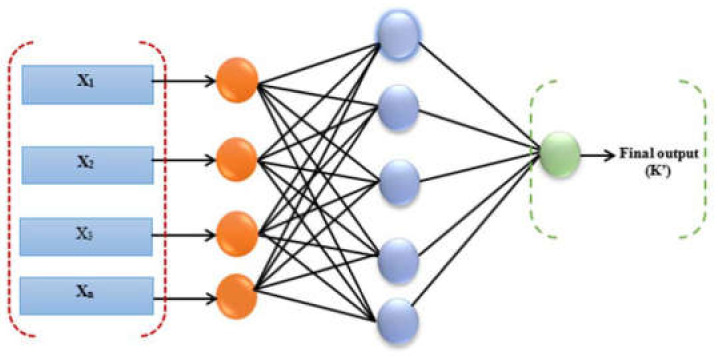
Architecture of ANN.

Artificial neural networks are machine learning algorithms that resemble the human brain in both physical and functional aspects. They link the layers of “neurons” (cells) that they employ to process and convey info. To assess whether a neuron should be triggered, the method uses a simple computation based on data from other neurons. The result of this computation is then passed to the neurons in the next layer [27]. The design of the three-layer feed-forward neural network used in this investigation is shown in Figure 1.

Neural networks normally only require a limited fraction of conventional mathematical operations; the formula for an artificial neural network will alter depending on the type of network employed to carry out a certain job. One of the most basic processes in neural networks is the dot product, which measures how similar two vectors are to one another. The following is the formula for the dot product of two vectors, x and w:

dot (x, w) = ∑x_i_* w_i_ (4)

where the total is computed over all members of the vectors, and x_i_ and w_i_ are the i-th and i-th elements of the x- and w-vectors, respectively [28].

Another common method applied to neural networks is the activation function, which is used to measure the results of the dot product in order to identify the output of a neuron. There are also numerous other activation functions that may be utilized, including the function of the sigmoid, the function of tanh, and the ReLU functions. The particular formula for the activation function will vary depending on the function being utilized. In this case, the sigmoid function can be expressed as

(5) $$f(x)=1/(1+e^{x})$$

which represents natural logarithm’s base (e) [28].

#### 2.3.3. Multiple Linear Regression (MLR)

The objective of MLR, a statistical approach, is to model the linear connection between a dependent variable and a group of independent factors. Given the values of the independent variables, the value of the dependent variable may be predicted. The dependent variable is represented in an MLR model as a linear mixture of the independent variables with an error feature that is thought to be random. Model parameters, or the coefficients of the independent variables, are computed using an optimization method like least squares. An MLR model can be represented generally as follows: Assuming y is the variable of dependence, x_1_, x_2_, ..., x_n_ are the variables of independence, b_0_, b_1_, ..., b_n_ are the model criteria, and e is the random error term, the formula is:

(6) $${y=b_{0}+b_{1}x_{1}+b_{2}x_{2}+...+b_{n}x_{n}+e}_{n}$$

MLR only uses one layer; it does not utilize neurons or intricate layer structures. The coefficients that are applied to each input parameter in MLR to determine how the parameter affects the output constitute the essence of the rules. The approach works for situations when the interaction among parameters is primarily linear, since it predicts the output by assuming a linear arrangement of inputs. As opposed to fuzzy logic or fuzzy inference systems, which use membership functions, MLR uses statistical concepts to estimate the coefficients and generate predictions depending on the inputs [29].

#### 2.3.4. Random Forest

The RF classifier is a combination of tree classifiers. The classifiers are built using random vectors that are extracted individually from the input vector, and the individual tree provides a unit preference for the most prevalent group to categorize a given input vector. Various decision trees are constructed in training using the Random Forest ensemble learning approach, which then integrates the predictions of the trees to produce reliable and accurate results. The approach makes use of the bagging technique, in which an initial sample of the initial dataset is used to train every tree. To increase variation, a randomly selected set of characteristic features is taken into consideration for dividing at every node in a tree. In tasks like classification, the final outcome is decided by an overwhelming vote of the trees, whereas in regression tasks, the average of the predictions made by each tree is used. In mathematical terms, the ensemble’s forecast (Y) for a novel input (X) is determined as follows if (T) is the set of trees of choice in the forest.

(7) for classification (Y=\text{mode}\{T_1(X),T_2(X),...,T_n(X)\}\)

(8) For regression, Y=\frac{1}{n}\sum_{i=1}^{n}T_i(X)\)

#### 2.3.5. Validation of Models

The main goal of statistical models is to adapt the framework to the available data in line with the indicators being employed to provide accurate forecasts for unknown datasets. The majority of the time, this is accomplished by changing the model to better fit the data. Overfitting is a concern because of this [30]. There are several alternatives for validation techniques, including k-fold, leave-one-out, cross-validation, holdout, and others. One such technique is cross-validation, which is sometimes referred to as k-fold cross-validation [31]. The holdout tactic is frequently considered to be more approachable than the intricate k-fold method. We split the gathered data into two samples, 50% for the training phase and 50% for the testing phase, considering the 4-fold cross-validation. It is important to remember that there are several methods for validating and segmenting the data.

#### 2.3.6. Model Performance Parameters

It is imperative to compare the projected values with the actual data obtained in order to assess how effectively a data-driven strategy worked. In this work, the models were assessed using a variety of statistical error metrics and a fit quality metric called the *R*^2^. Other metrics utilized were the *R*, MSE, MAPE, and RMSE [32]:

(9) $$R^{2}=1-\frac{\sum_{j=1}^{N}{\left[{\left(Y\right)}_{obs,j}-{\left(Y\right)}_{com,j}\right]}^{2}}{\sum_{j=1}^{N}{\left[{\left(Y\right)}_{obs,j}-{\bar{\left(Y\right)}}_{obs,j}\right]}^{2}}$$

(10) $$R=\frac{\sum_{i=1}^{N}\left(Y_{obs,i}-{\bar{Y}}_{obs,i})(Y_{com,i}-{\bar{Y}}_{com,i}\right)}{\sqrt{\sum_{i=1}^{N}{\left(Y_{obs,i}-{\bar{Y}}_{obs,i}\right)}^{2}\sum_{i=1}^{N}{\left(Y_{com,i}-{\bar{Y}}_{com,i}\right)}^{2}}}$$

(11) $$\mathrm{MSE}=\frac{1}{N}\;\sum_{i=1}^{N}(Y_{obs,i}-Y_{com,i}{)}^{2}$$

(12) $$RMSE=\sqrt{\frac{\sum_{i=1}^{N}{\left(Y_{obs,i}-Y_{com,i}\right)}^{2}}{N}}$$

where *N* is the aggregate amount of points in the dataset, *Y_obs_* is the total number for observed data points, *Y* is the mean value of the data that was observed, and *Y_com_* is the computed value.

## 3. Results 

### Methodological Procedure

**Figure 2:**
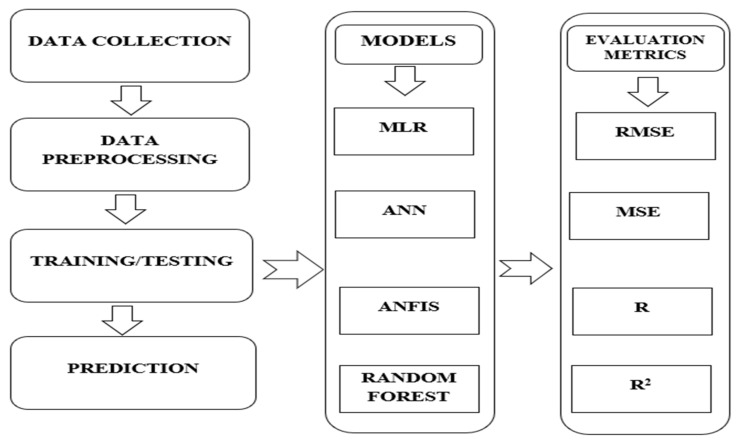
Experimental set-up.

The methodical procedure of data collection, data preparation, model training, testing, and prediction comprised the experimental setup for our investigation. Furthermore, the MLR, ANN, ANFIS and Random Forest models are adopted as our machine learning classifiers. The results are evaluated using the evaluation metrics, RMSE, MSE, *R* and *R*^2^, as shown illustrated in Figure 2.

The malaria parasite was predicted using associated independent factors and data-driven approaches including MLR, ANN, ANFIS and Random Forest. Table 4 shows the findings of the statistical analysis of the data before going into further depth about the model calibration. Data analysis helps identify the data’s scientific and navigational worth, resolving issues that could otherwise prohibit correct simulation of the outcomes. The model that was created and then utilized to build the ANN, ANFIS and Random Forest models was created using MATLAB 9.3 (R2019A). Correlation studies were performed using Microsoft Excel (Microsoft Excel Professional plus 2019. Version 1808 (Build 10405.20015)), which was also used to create the traditional linear regression (MLR). It was decided to take the average of the segmented, data-driven correlations of the 15 input variables.

The models’ performance metrics during the training stage demonstrate that ANN fared better than the others, showing the least RMSE (0.000906) and MSE (0.000905) as well as the greatest *R*^2^ (0.999661) and *R* (0.99983). ANFIS performed admirably as well, displaying an *R*^2^ of 0.947893 and *R* of 0.973598. MLR was relatively less precise, yet it produced findings that were still rather good. But in testing, Random Forest fared better than the other models, exhibiting the lowest RMSE (0.0583) and MSE (0.2414) and the highest *R*^2^ (0.7648) and *R* (0.8752). This suggests that while Random Forest was revealed to be the most reliable through the testing phase, ANN performed exceptionally well throughout the training phase. As a consequence, the effectiveness of the results from the current study is consistent with studies about the present and future status of machine learning models in identifying malaria parasites published by Ozsahin et al. Their method has made it feasible to swiftly comprehend the machine learning. The effectiveness of the models was assessed during the testing phase and training phase.

**Figure 3:**
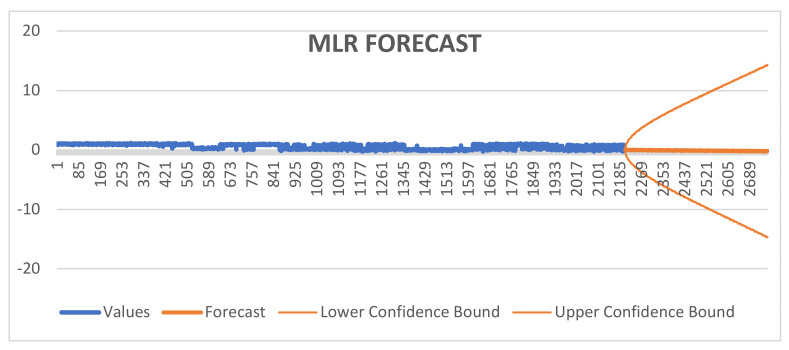
Forecasting MLR.

The forecast sheet has a variety of statistical metrics that show how well and accurately a forecasting model performs. RMSE, MASE, SMAPE, Alpha, Beta, and Gamma data are shown. The Alpha value of 0.75 indicates a rather high level of confidence in the statistical study. When the beta value is 0, it means that there is no evidence of a Type II error and that the model is not missing any significant trends or components. In a manner similar to the preceding illustration, a Gamma value of 0.00 shows that there is no evidence of a Type I error, demonstrating that the model is not incorrectly identifying any significant components. The MASE rating of 1.74 denotes the forecast’s accuracy in relation to the scale, as shown in Figure 3.

SMAPE score of 0.98 calculates the percentage difference between the expected and actual values. A forecast error’s average size is represented by the MAE, which has a value of 0.30. Taking into consideration the squared values of forecast errors, the RMSE, which represents the average size of forecast errors, has a value of 0.40. The precision and accuracy of the projections generated by the forecasting model are essential topics covered by these statistics overall, as shown in Table 5.

## 4. Discussion

Employing a sample of 2207 patients, the findings of this research offer insightful information about how well various machine learning algorithms predict malaria. ANN showed outstanding precision throughout the training phase, attaining the greatest *R*^2^, *R*, and least RMSE and MSE across the models. This demonstrates how ANNs can efficiently identify intricate associations in the data while they are learning. Conversely, the ANFIS demonstrated a high level of performance, demonstrating its adeptness in managing the dataset. Although it produced acceptable results, MLR was relatively less accurate when it was being trained. Interestingly, Random Forest performed better than the other algorithms having the highest *R*^2^ and *R*, as well as the least RMSE and MSE, and it was considered the most stable predictor throughout the testing stage. This demonstrates Random Forest’s capacity to generalize and generate precise predictions on novel, untested datasets. Random Forest’s testing phase efficiency was better than expected, indicating that it can handle real-world events and generalize effectively to a variation of datasets.

To evaluate the effectiveness of the model for prediction, the forecast sheet included a number of statistical indicators, such as RMSE, MASE, SMAPE, Alpha, Beta, and Gamma. While a Beta value of 0 reveals no evidence of a Type II mistake, suggesting that major patterns or components are not neglected, a high Alpha score of 0.75 reflects a high degree of certainty in the statistical investigation. A Gamma value of 0.00, on the other hand, implies that there is no proof of a Type I error and that the model accurately detects key components. The forecast’s accuracy in relation to the scale is indicated by the MASE score of 1.74.

The percentage difference of predicted and actual data is derived using an SMAPE score of 0.98. The MAE indicates the mean size of forecast mistakes in this scenario, which is 0.30. The RMSE reflects the average size of forecast errors and has a value of 0.40 when the squared values of the errors in forecasting are considered. These quantitative metrics, as shown in Table 5, represent the prediction model’s total ability to provide projections with clarity and accuracy.

Comparatively, this study is distinctively outstanding when compared with other studies such as the study by [4]. The authors adopted a single machine learning model, the ANN in their study, which recorded 85%, which was lower than our prediction with 99% in prediction. Similarly, there is no similar study that adopted ANN, MLR and ANFIS in the prediction of a malaria parasite dataset with similar evaluation metrics. Similarly, this study is unique due to the adoption of both statistical and machine learning approaches in the analysis.

Multi-linear regression utilizing geographic information systems (GIS) and satellite imagery was employed to map the spread of malaria in India’s Varanasi district. The dependent variable in the study by [14] was malaria cases recorded in the research location, and the independent factors were several time-based groupings of average temperature data. To build a malaria susceptibility map for both qualitative and quantitative characteristics, a 50 × 50 sample was translated from GIS to statistical software. However, in [14], the authors used both GIS and statistical approaches in their research.

Our study achieves exceptional results by utilizing sophisticated machine learning techniques, specifically artificial neural networks (ANNs), to forecast malaria occurrence. It effectively analyzes a large dataset with various variables, producing amazing disease prediction accuracy. The study by [14], on the other hand, uses multi-linear regression combined with GIS and remote sensing to track malaria spread in a particular area. While the study sheds light on the effect of temperature on instances of malaria, our study’s extensive use of machine learning and broader variable coverage demonstrates superior predicting capabilities, thereby rendering it flexible and efficient for tackling malaria-related issues.

Furthermore, our study outperforms the study by Ozsahin et al., which concentrated on malaria parasite detection employing deep learning techniques. A range of predictive models, comprising artificial neural networks (ANNs), adaptive neuro-fuzzy inference systems (ANFISs), and multi-linear regression (MLR), were used in our study, allowing for a full examination of a diversified dataset with various variables. This resulted in extremely accurate disease forecasts. In contrast, Ozsahin et al. focused primarily on deep learning for malaria parasite detection, emphasizing the selection of the best blood smear type. While their convolutional neural network demonstrated remarkable accuracy, precision, and sensitivity, our study’s comprehensive use of machine learning models provided a more comprehensive and flexible approach to addressing malaria-related difficulties, making the other study’s findings more compelling.

When compared to Yadav et al.’s study, which similarly addresses machine learning-based malaria prediction, our study outperforms them. While Yadav et al. applied a variety of machine learning techniques to forecast malaria based on clinical data, the first study distinguishes itself by employing a wider range of machine learning models, which includes artificial neural networks (ANN), adaptive neuro-fuzzy inference systems (ANFISs), and multi-linear regression (MLR). This comprehensive strategy yields extremely precise disease forecasts as well as variable coverage, indicating its superior effectiveness in solving malaria-related difficulties.

In conclusion, the study highlights how crucial it is to take testing and training performance into account when assessing machine learning algorithms. ANN performed very well in training, while Random Forest performed better in testing in terms of resilience and generalization. These results add to the continuing discussion about which models are best for predicting malaria with ramifications for the distribution of resources and healthcare choices. Subsequent investigations may investigate group techniques or combined models to use the advantages of several algorithms for improved prognostic precision in malaria cases.

This study has a few drawbacks that should be mentioned. To begin, the study focused solely on machine learning methods for detecting malaria parasites, perhaps overlooking other relevant factors. Second, due to the unique dataset used, the findings may not be broadly applicable, limiting generalizability. Finally, the comparison was limited to MLR, ANN, ANFIS and Random Forest models, with no additional approaches considered. These constraints highlight the need for more research to close gaps and enhance the accuracy and usefulness of malaria prediction systems.

## 5. Conclusions

The results highlight how important it is to take algorithm performance into account in both learning and real-world contexts. The particular needs and features of the dataset may influence the best prediction model selection. These revelations advance our knowledge of machine learning uses in healthcare as it relates to malaria predictions. Investigating hybrid models or ensemble techniques as future research could improve prediction accuracy even more. In summary, this research offers significant insights for medical professionals and legislators, enabling well-informed choices about the control of malaria and the distribution of available resources.

## Figures and Tables

**Table 1:** Epidemiology of malaria parasite [9].

Rank	Country	Cases (Estimated)
1	Nigeria	94,000,000
2	Democratic Republic of the Congo	16,000,000
3	Mozambique	10,400,000
4	India	6,500,000
5	Uganda	5,700,000
6	Burkina Faso	4,300,000
7	Niger	3,800,000
8	Malawi	3,400,000
9	Mali	3,100,000
10	Tanzania	3,000,000

**Table 2:** Parameters and values.

Features	Data Type	Value (Binary)
Fever Symptom	Independent	0 (negative) or 1 (positive)
Temperature	Independent	
Rapid Diagnostic Test (RDT)	Independent	0 (negative) or 1 (positive)
White Blood Cell Count (WBC),	Independent	0 (negative) or 1 (positive)
Red Blood Cell Count (RBC)	Independent	0 (negative) or 1 (positive)
Hemoglobin Level	Independent	0 (negative) or 1 (positive)
Hematocrit	Independent	0 (negative) or 1 (positive)
Mean Cell Volume	Independent	0 (negative) or 1 (positive)
Mean Corp Hb	Independent	0 (negative) or 1 (positive)
Mean Cell Hb Conc	Independent	0 (negative) or 1 (positive)
Platelet Count	Independent	0 (negative) or 1 (positive)
Platelet Distribution Width	Independent	0 (negative) or 1 (positive)
Neutrophils Percent	Independent	0 (negative) or 1 (positive)
Lymphocytes Percent	Independent	0 (negative) or 1 (positive)
Mean Platelet Volume	Independent	0 (negative) or 1 (positive)
Mixed Cells Percent	Independent	0 (negative) or 1 (positive)
Neutrophils Count	Independent	0 (negative) or 1 (positive)
Lymphocytes Count	Independent	0 (negative) or 1 (positive)
Mixed Cells Count	Independent	0 (negative) or 1 (positive)
RBC Distribution Width Percent	Independent	0 (negative) or 1 (positive)
Microscopy	Dependent	0 (negative) or 1 (positive)

**Table 3:** ANN modeling summary.

ANN Architecture		
Type	Feed-forward back-propagation	
Number of layers	15	
	Input layers	14
	Hidden layers	15
	Output layers	1
Training parameters	Training method	Supervised
	Training algorithm	Malaria data
	Training data	70% of all the data
Activation function	In hidden layer	Sigmoid
	In output layer	Linear transfer function
Epochs	6	
Testing data	Amount of data	30% of all data

**Table 4:** Result of the models.

	TRAINING					TESTING			
	MLR	ANFIS	ANN	Random Forest		MLR	ANFIS	ANN	Random Forest
*R* ^2^	0.848586	0.947893	0.999661	0.6873	*R* ^2^	0.951865	0.967739	0.974009	0.7648
*R*	0.921187	0.973598	0.99983	0.8358	*R*	0.975636	0.983737	0.986919	0.8752
RMSE	0.000834	0.000882	0.000906	0.0775	RMSE	0.000883	0.00089	0.000893	0.0583
MSE	0.000769	0.000859	0.000905	0.2783	MSE	0.000861	0.000876	0.000881	0.2414

**Table 5:** Statistics of the forecast.

Statistics	Value
Alpha	0.75
Beta	0.00
Gamma	0.00
MASE	1.74
SMAPE	0.98
MAE	0.30
RMSE	0.40

## Data Availability

The data is available upon the requests from the authors and it is shared in an open source platform which is cited in the article.
